# Conceptual framework and uncertainty analysis for large-scale, species-agnostic modelling of landscape connectivity across Alberta, Canada

**DOI:** 10.1038/s41598-020-63545-z

**Published:** 2020-04-22

**Authors:** Ronan Marrec, Hossam E. Abdel Moniem, Majid Iravani, Branko Hricko, Jahan Kariyeva, Helene H. Wagner

**Affiliations:** 1 Department of Ecology and Evolutionary Biology, University of Toronto, Toronto, Ontario Canada; 2 EDYSAN (Ecologie et Dynamique des Systèmes Anthropisés) UMR 7058 CNRS-Université de Picardie Jules Verne, 33 rue Saint Leu, F-80039 Amiens, France; 3 Centre for Urban Environments, University of Toronto Mississauga, Mississauga, Ontario Canada; 4 Department of Zoology, Faculty of Science, Suez Canal University, Ismailia, Egypt; 5 Alberta Biodiversity Monitoring Institute, University of Alberta, Edmonton, Alberta Canada

**Keywords:** Biogeography, Community ecology

## Abstract

Sustainable land-use planning should consider large-scale landscape connectivity. Commonly-used species-specific connectivity models are difficult to generalize for a wide range of taxa. In the context of multi-functional land-use planning, there is growing interest in species-agnostic approaches, modelling connectivity as a function of human landscape modification. We propose a conceptual framework, apply it to model connectivity as current density across Alberta, Canada, and assess map sensitivity to modelling decisions. We directly compared the uncertainty related to (1) the definition of the degree of human modification, (2) the decision whether water bodies are considered barriers to movement, and (3) the scaling function used to translate degree of human modification into resistance values. Connectivity maps were most sensitive to the consideration of water as barrier to movement, followed by the choice of scaling function, whereas maps were more robust to different conceptualizations of the degree of human modification. We observed higher concordance among cells with high (standardized) current density values than among cells with low values, which supports the identification of cells contributing to larger-scale connectivity based on a cut-off value. We conclude that every parameter in species-agnostic connectivity modelling requires attention, not only the definition of often-criticized expert-based degrees of human modification.

## Introduction

Land-use and land-cover changes have impacted many natural ecosystems to provide ecosystem goods and services for an ever-growing human population^1^. This often has unintended consequences that may threaten biodiversity and ecosystem health^2^. Such consequences include changes in local, regional, and global climate^3^, alteration of natural habitats^4^, pollution of land, air, and water^5–7^, and changes in landscape structure, i.e., the total area and spatial configuration of ecosystems^8^.

Natural ecosystems offer habitat for many species, and human landscape modification typically involves habitat loss as well as the breaking up of continuous habitats into smaller remnant patches (fragmentation)^9,10^. Consequently, landscapes may lose connectivity, i.e., the degree to which they facilitate movement of organisms and their genes among patches^11,12^. Landscape connectivity can be quantified in three ways: structural landscape connectivity, potential functional connectivity, and actual functional connectivity^13^. Structural landscape connectivity can be determined from physical attributes, based on maps alone without reference to organismal movement behaviour. Potential functional connectivity relies on a set of assumptions on organismal movement behaviour to implement an organism perspective, e.g. by mapping a species’ habitat and setting a dispersal threshold. In contrast, actual functional connectivity refers to observed data (e.g., patch occupancy, radio tracking, mark-recapture, or molecular genetic data) that reflect actual rates of the exchange of individuals (or their genes) and may be used to test models of structural or potential functional connectivity^13^. As landscape connectivity facilitates organism dispersal, gene flow, and many other ecological functions of a landscape^14^, its erosion is a major concern for wildlife population survival, due to increase of extinction risk, loss of species diversity, and disruption of major ecosystem services^11,15–17^. For these reasons, connectivity is considered a key aspect of land-use planning and conservation management^18,19^.

Connectivity maps are sensitive to the way connectivity models are conceptualized and implemented^20,21^, and there is no general consensus on which approach should be used preferentially to support planning. The most common approach to modelling connectivity is the focal species approach^10,22^. This bottom-up approach considers one or a few species that serve as surrogates to characterize connectivity for a larger suite of species^23^. It aims to evaluate the potential functional connectivity of a species’ habitat by taking into account species-specific dispersal thresholds and modelling the impeding effect of different feature types on movement as resistance values^24^. However, because species differ, e.g., in habitat requirements, body size, dispersal ability, or lifespan, the effect of habitat fragmentation on functional connectivity can differ importantly^25,26^. For this reason, the effectiveness of such an approach to evaluate connectivity for a suite of species is highly debated^27–29^. In addition, large-scale analyses may require large numbers of focal species to represent diverse habitat types^20^. To overcome these limitations, there is growing interest in applying a top-down approach, which does not rely on biological or ecological characteristics for specific taxa to model large-scale connectivity maps for management and planning efforts^30,31^, recently known as a “species-agnostic” approach (e.g.^32^). More specifically, these models are solely based on quantifying the degree of unnaturalness in the landscapes which are caused by human modifications^29^ as well as other ecological and geochemical processes. Indeed, the approach is based on the assumption that natural terrestrial areas facilitate connectivity, and the higher the degree of human modification, the more the ‘ecological flow’ is restricted. Models based on structural connectivity are not new. For instance, effective mesh size^30^ likewise does not make reference to species characteristics. It is important to note that even a species-agnostic approach will be parameterized with a certain group of species in mind, in this case terrestrial organisms^20,33^.

Because large water bodies, despite being natural features, may disrupt the movement of many terrestrial organisms, some authors have treated them as a barrier^34^. The hydrological connectivity of aquatic habitats, on the other hand, should be modelled separately as it has a linear network structure.

More generally, focal-species and species-agnostic approaches both are known to be sensitive to resistance values, which are often based on expert opinion, and a rigorous assessment of the sensitivity to parameter settings is required^35–37^. For instance, Koen *et al*.^28^ successfully validated a species-agnostic model of landscape connectivity with road mortality data of reptiles and amphibians and with molecular genetic data for a mammalian species. Connectivity models are also known to be sensitive to the degree of contrast between high-resistance and low-resistance landscape features, which can be modified with a scaling function^38,39^. Moreover, Arponen *et al*.^40^ demonstrated that the spatial resolution of large-scale connectivity maps has an influence on the prioritization of areas for conservation. Nevertheless, parameterization and optimization of resistance surfaces in a biologically and ecologically relevant way for functional connectivity across species remains a nontrivial challenge^41^, and a better understanding of the relative importance of these factors is needed. Given a resistance surface, connectivity can be evaluated either by least cost path analysis^24^ or by quantifying current density based on circuit theory^32,42^. The latter approach is commonly used in landscape ecology and genetics studies^32,42^ as it allows for multiple movement pathways and varying degrees of corridor use across the landscape^43^. This makes it possible to investigate multiple corridor routing options^44^.

The spatial resolution of resistance maps has been shown to affect the accuracy of resulting connectivity maps through its effect on many landscape pattern metrics (e.g.^45–47^), including connectivity metrics^48^. Indeed, increasing spatial resolution (grain size) has a large effect on the accuracy of circuit-based connectivity estimates^37^. However, until recently^49^, computational limitations have precluded the use of circuit theory models to compute connectivity for large-extent, high-resolution maps^29,50^. Consequently, wall-to-wall, large-scale, and fine-grain computation of connectivity maps using circuit theory remains largely understudied, despite of increasing demand and recent expansions of this modelling approach^10,28,34,51^. Recent computational advances, namely the availability of GFlow^49^ and the new implementation of Circuitscape^52^ in Julia, have overcome previous computational limitations of Circuitscape and provide an important opportunity for developing and testing large-scale connectivity models based on current density.

Here we (*i*) propose a conceptual framework for species-agnostic connectivity modelling based on current density; (*ii*) discuss conceptual and computational decisions involved in implementing the approach; and (*iii*) assess the degree of uncertainty related to these decisions. The applied goal is to derive one main wall-to-wall map of current density as a measure of species-agnostic connectivity for Alberta based on the degree of human modification. The province of Alberta, Canada, is aiming to integrate an index of landscape connectivity into their biodiversity and ecosystem services assessment framework^53^. The Alberta Biodiversity Monitoring Institute (ABMI) is systematically collecting data on both human footprints and biodiversity for the province of Alberta at a level of detail, spatial extent, and spatiotemporal resolution that are unique at least for North America^54^ (see also the ABMI’s 10-year Science and Program Review, https://abmi10years.ca). Alberta, with a total area of 661,848 km^2^, has a human population of over 4 million (2016 census) and one of the biggest economies in Canada, including an expanding petroleum industry and significant contributions from agriculture, forestry, and tourism^55^. Because of rapid growth in population and economic activity, Alberta’s landscapes have changed noticeably over recent decades, and the rate of development is expected to increase further^56^, adding unprecedented pressure on remaining natural areas and low-intensity land uses^57,58^. Alberta defined a Land-use Framework^53,59^ to manage and sustain a growing economy, while balancing this with Albertan’s social and environmental goals. To improve land-use planning and biodiversity management in Alberta, policy makers, landowners, and other stakeholders are thus in need of new tools to evaluate the ecological value of ecosystems and landscapes, including an assessment of the contribution of an area to larger-scale connectivity, so as to compare the impact of alternative development scenarios and prioritize areas for conservation and restoration.

After deriving a main wall-to-wall map of current density as a measure of species-agnostic connectivity for Alberta based on the degree of human modification, we use this example to compare the contributions of different factors to uncertainty. We thus assess the sensitivity of this map to (*i*) the definition of the degree of human modification (i.e., the relative weight given to degree of physical human footprint *vs*. intensity of human use), (*ii*) the decision whether water bodies are considered barriers to movement, and (*iii*) the scaling function used to translate degree of human modification into resistance values. While the influence of most of these parameters on connectivity patterns has been previously studied independently, we aim to directly compare their contributions to uncertainty. We further (*iv*) evaluate the effect of such uncertainty on the classification of cells as being important or less important for maintaining larger-scale connectivity using different cut-off values. This study prepares the ground for future research to evaluate model validity with Alberta’s extensive biodiversity monitoring data.

### Conceptual framework

**Figure 1:**
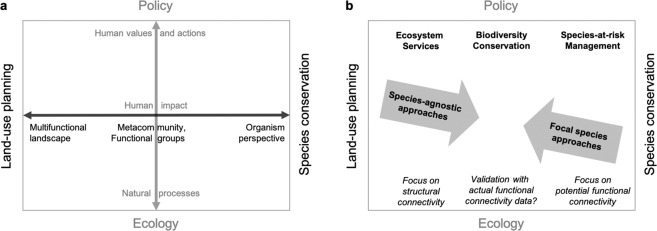
Conceptual framework: different perspectives on the landscape (**a**) and corresponding goals and approaches to connectivity modelling (**b**). The horizontal axis (black) illustrates how the landscape definition shifts from a multi-functional landscape in the context of land-use planning to an organism perspective in the context of species conservation. The vertical axis (grey) illustrates how the focus shifts from natural processes in basic ecology to human values and actions in a policy context. These values are reflected in policy that aims to preserve ecosystem services, biodiversity in general, or designated species of concern. Species-agnostic modelling of landscape connectivity may be most suitable in a land-use planning context, whereas focal species approaches may be most suitable in a conservation management context. When biodiversity data are available for many taxa, multi-species overlay can also be an efficient option to model connectivity for land-use planning and conservation programs for a representative suite of species. Validation of structural connectivity or potential functional connectivity based models could also be done with appropriate biological data, to assess actual functional connectivity.

We argue that a species-agnostic approach is conceptually better suited for integrating landscape connectivity into land-use planning, whereas a focal-species approach is better suited for conservation management (Fig. 1). Understanding the difference between these perspectives can help clarify conceptual differences, guide researchers in making decisions about how to model connectivity, and inform practitioners about the potential and limitations of resulting maps. Conservation is often based on focal species, e.g., in species-at-risk management, where it is paramount to adopt an organism perspective^60^ to define critical habitat and to consider the organism’s ability to move between habitat patches. This will result in a model of potential functional connectivity for the specific organism of interest, and a multi-species model can be derived by overlaying models for a representative suite of species^10,22^. In contrast, land-use planning focuses on the sustainable development of multi-functional landscapes. In this human-centred perspective, land parcel ownership and administrative boundaries define the relevant spatial scale and the degree to which landscape development can be influenced by policy, which in the case of Alberta includes the introduction of ecosystem services and biodiversity markets that play a major role in balancing environmental considerations with socio-economic drivers^59^. Regarding landscape connectivity, the focus thus lies on how human landscape alteration affects the connectivity of the remaining natural heritage system and how to compare the expected effect of local development alternatives on larger-scale connectivity. This focus is highly compatible with the modelling of connectivity based on human modification, which will result in a model of structural landscape connectivity.

A coarse-filter approach to biodiversity conservation management falls somewhere in-between^61,62^. The focus in this approach lies on preserving a community, such as grassland, wetland, or forest interior species, by maintaining a sufficient amount, quality, and connectivity of the respective ecosystem in the landscape. This implies that the species within a community have similar needs and characteristics, including their ability to move between habitat patches. Regarding landscape connectivity, we suggest that species-level data, such as mark-recapture, radio-telemetry, and molecular genetics data, which provide a direct measure of actual functional connectivity, may be used to test and compare species-agnostic (top-down) or multi-species (bottom-up) connectivity models. At the community-level, however, data such as biodiversity monitoring that is systematically collected across a large geographic area can be used as indirect measures of functional connectivity. If a connectivity model can explain, e.g., variation in species composition that remains unexplained by local site characteristics, this may indicate that it successfully captured the shared response of a broad range of species to human landscape modification. Note that there is a lack of empirical studies that compare a general-use connectivity assessment based on the overlay of many species-specific models across taxonomic groups to a species-agnostic model.

Another distinction occurs along the vertical axis in Fig. 1a: while ecology primarily considers natural processes, policy addresses human values and actions. The two perspectives meet in the consideration of the human impact on ecosystems. Based on this framework, a species-agnostic approach to modelling landscape connectivity quantifies how human actions and their manifestation as non-natural landscape features constrain ecological flow across the underlying natural fabric of the landscape. The main goals of this approach are to identify critical linkages and to support the evaluation of alternative development scenarios, with the general aim of maintaining ecosystem services and biodiversity in the context of sustainable development. This cannot replace fine-filter approaches for specific conservation goals, such as species-at-risk management. Note also that ecosystems can have an impact on humans as well, such as the spread of animal-transmitted diseases (e.g., West-Nile virus or Lyme disease). Here, we focus on human impacts on natural ecosystems.

To implement a species-agnostic approach, resistance values are assigned to non-natural landscape features based on expert opinion^33^. Conceptually, this involves two steps: (1) quantifying the degree of human modification, and (2) using a scaling function to assign resistance values based on the degree of human modification. We argue that beyond an inevitable element of subjectivity in expert-defined values, different interpretations of what constitutes degree of human modification can result in considerable differences between values. For instance, two types of roads may have the same physical footprint in terms of deviation from natural conditions (impermeable surface) but differ vastly in their intensity of use (traffic volume), which will affect e.g. road mortality rates. The relative scaling of resistance values is known to have a large effect on connectivity modelling, and a sharp contrast between low, medium or high resistance values has previously been suggested and used^34,38,39^.

In addition to human modification, some authors including Dickson *et al*.^34^ also considered natural barriers to the movement of terrestrial organisms and applied resistance values to features like water bodies and topography. Conceptually, this introduced an element of potential functional connectivity as it makes assumptions about the movement ability of organisms. In practice, this raises the concern that, e.g., water bodies may be traversable for some terrestrial species but not for others. Specifically, water bodies are a special case in such a framework as they are not a terrestrial feature, and while they are mostly natural, some authors have treated them as a barrier. However, in the context of species-agnostic models, much less thought has been given to the decision how to conceptualize water bodies than to other factors.

Here, we define a conceptually justified range of variation for the three factors identified above, with the goal of directly comparing their relative importance, as they have been addressed independently in previous studies on species-agnostic connectivity modelling: (*i*) the assignment of degree of human modification values by degree of deviation from natural conditions (footprint) or by the intensity of use; (*ii*) the assignment of resistance value to water bodies between minimum (natural) and a value close to the maximum of all human features combined (near-complete barrier); and (*iii*) the choice of scaling function resulting in a weak or strong contrast between non-natural and natural landscape features. We use intermediate values^33^ to create the main map, and we assess uncertainty in a factorial design using the extremes of each factor.

Circuit theory models connectivity as current density, where areas with high current density are interpreted as contributing to larger-scale landscape connectivity and thus ‘ecological flow’ across the study area^32,42^. We thus assess the contribution of each factor to variation among maps in the quantification of current density as the contribution of a cell to large-scale connectivity. We argue that visual interpretation of current density maps is affected by the choice of colour ramp, which should reflect a conscious decision about how cells with important contribution to large-scale connectivity (high current density values) are identified. J. Bowman (Trent University, *pers. comm*.) suggested that visualizing and interpreting current density values that have values higher than one standard deviation above the map mean (i.e., *z* scores> 1) as contributing areas provides a good balance for application. Here, we further vary this cut-off between the mean (i.e., *z* scores> 0) and two standard deviations above the mean (i.e., *z* scores> 2) to assess whether the uncertainty related to the factors above varies with the cut-off level used.

## Results

**Figure 2:**
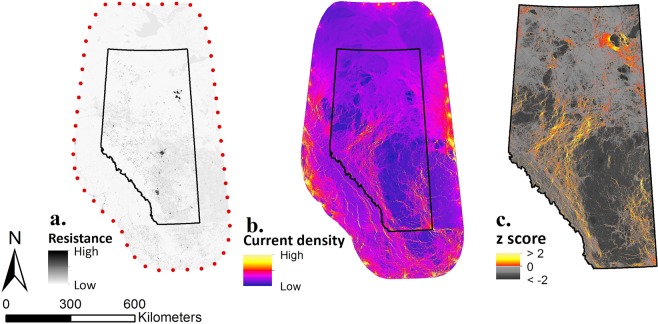
Main map computation pipeline. Main map was calculated using an average combination (*H*_*FU*_) of physical human footprint, *H*_*F*_, and intensity of human use, *H*_*U*_, resistance values. Map **(a)** shows resistance map calculated at 100 m resolution where darker values represent high resistance and brighter values represent lower resistance (red dots, nodes used to compute the following current density map). The map was computed in R 3.4.2^83^ and QuantumGIS^80^. Map **(b)** is the current density map resulting from GFlow’s^49^ electric current simulation at a 100 m resolution. Red to yellow colour ramp represents higher values of current density while darker purple to blue colour ramp represents lower values of current density. Map **(c)** is clipped to Alberta extent and current density values were standardized and centred at a 100 m resolution. Hot colour ramp represents important connectivity cells with a *z* > 0, and grey scale colour ramp represents unimportant connectivity cells with *z* < 0. The map was computed in R 3.4.2^83^.

The main map (Fig. 2) shows prominent connectivity pathways extending mainly from the southwest to the northeast of Alberta. More specifically, areas of high current density are concentrated in the foothill region, connecting the Rocky Mountain range to the forest-dominated northern part of the province. Patterns of high current density were also observed in the south-eastern grassland and in the north-eastern Canadian Shield region of the province.

**Figure 3:**
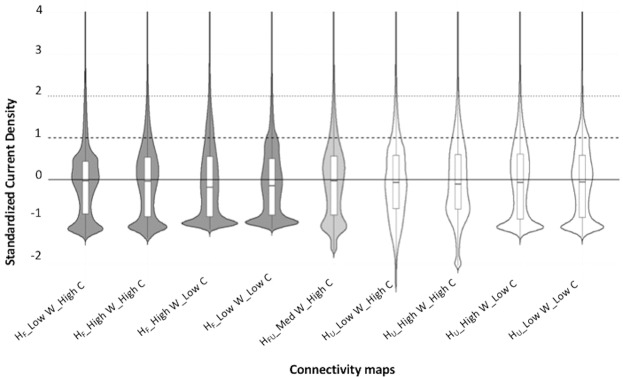
Cell distribution following their scaled (with mean = 0) current density in the four *H*_*F*_ maps (dark grey), the four *H*_*U*_ maps (white), and the main, *H*_*FU*_ map (light grey). Violin plot width is proportional to cell density. The boxplots show median and quartiles of each distribution. The *z*-score cut-offs are represented as horizontal lines: *z* = 0 (solid), 1 (dashed), and 2 (dotted).

**Figure 4:**
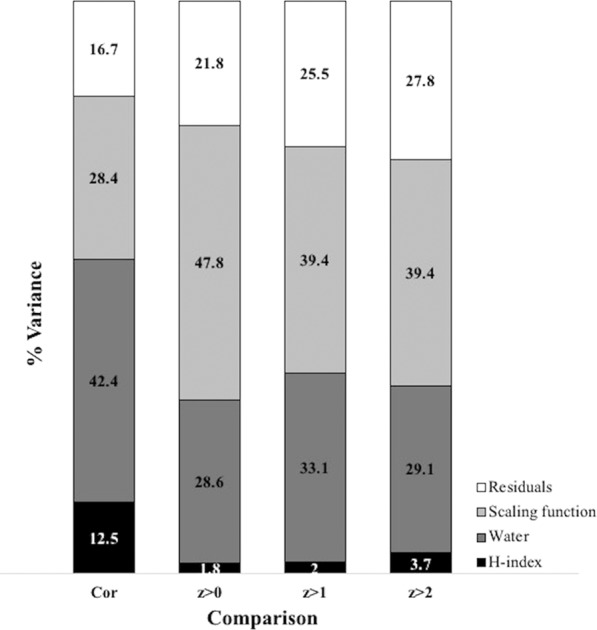
Variance partitioning analysis depicting relative contribution of each choice factor: *H* index (either *H*_*F*_ or *H*_*U*_; black); water resistance (either 0 or 1000; dark grey); and scaling function (either linear or derived power; light grey). Analysis was done separately for each *z*-score cut-off used to identify important cells, (either *z* = 0, 1, or 2) and for the cell-by-cell correlations in current density values between maps (Cor). Variances are presented as percentages (%).

The uncertainty analysis showed large variations in absolute current density values among maps, both in their ranges (Table 1) and distributions (Fig. 3). Differences in the shape of distribution of current density values were most pronounced for values below the mean. The proportion of cells with *z* > 1 (or *z* > 2) was quite constant, but more variable for cells with *z* > 0 (Fig. 3). These variations resulted in different patterns of high current flow pathways between maps (Fig. 4).

**Table 1:** Resistance and current density ranges and standard deviations of the eight maps used in the uncertainty analysis (*H*_*F*_ and *H*_*U*_ maps) and the main, average map (*H*_*FU*_ map).

*H* index	Water resistance	Scaling contrast	Resistance		Current Density	
			Range	SD	Range	SD
*H*_*F*_	Low	High	1–1042.457	145.781	~0.0–0.274	0.0035
	High	High	1–2032.041	238.583	~0.0–0.418	0.0037
	High	Low	1–1066.741	296.91	~0.0–0.184	0.0036
	Low	Low	1–1019.457	297.878	~0.0–0.333	0.0031
*H*_*FU*_	Medium		1–1089.033	84.43	~0.0–0.137	0.0035
*H*_*U*_	Low	High	1–1042.457	75.434	~0.0–0.087	0.0027
	High	High	1–2031.333	207.989	~0.0–0.448	0.0029
	High	Low	1–1066.033	145.567	~0.0–0.182	0.003
	Low	Low	1–1019.457	146.282	~0.0–0.212	0.0031

**Figure 5:**
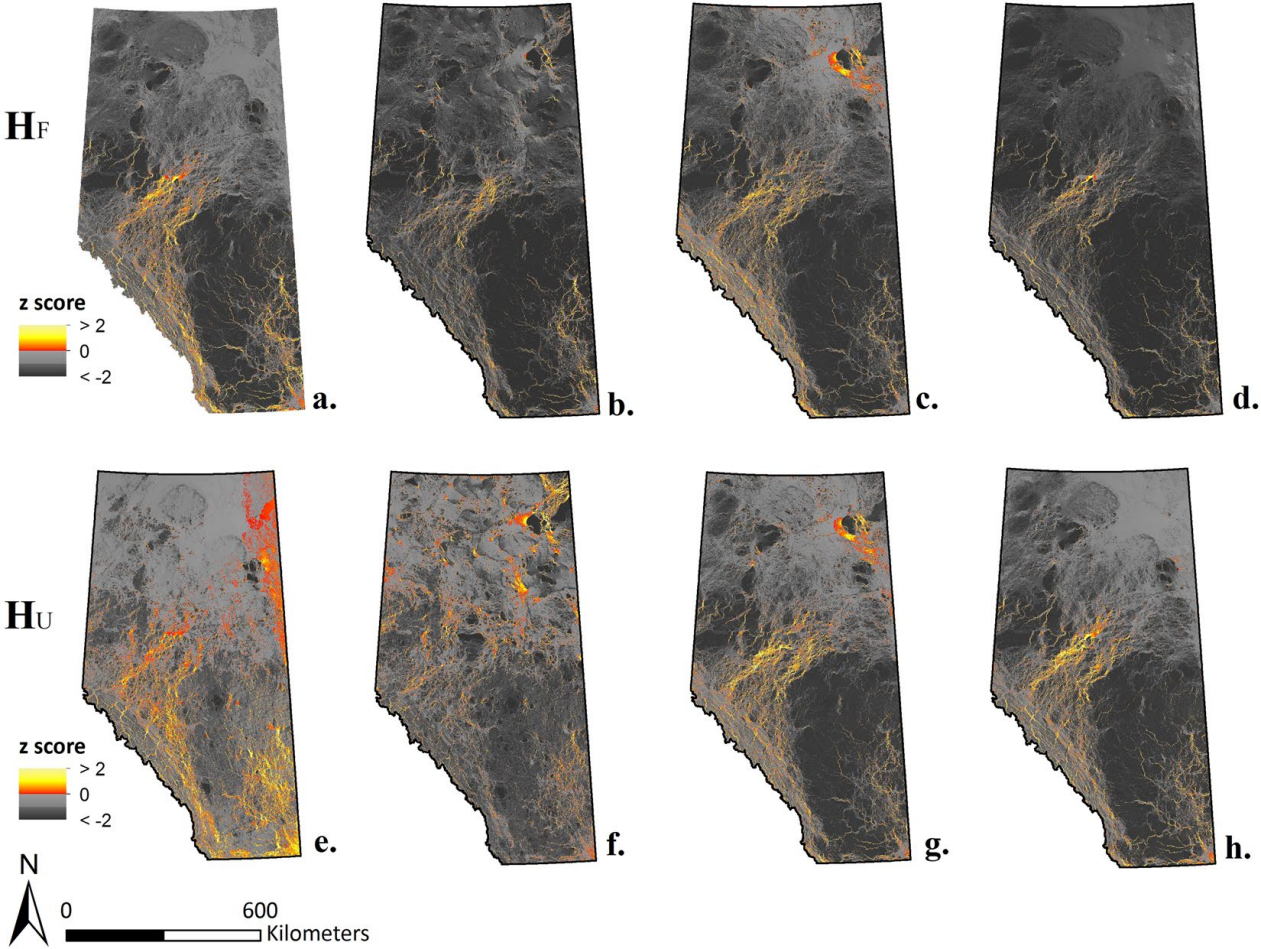
Scaled (with mean = 0) current density maps of Alberta at 100 × 100 m resolution using either *H*_*F*_ **(a–d)** or *H*_*U*_ **(e–h)** as human modification index. *H* index was considered in combination with: minimum water resistance and maximum scaling **(a,e)**; maximum water resistance and maximum scaling **(b,f)**; maximum water resistance and minimum scaling **(c,g)**; and minimum water resistance and minimum scaling **(d,h)**. Hot colour ramp represents important connectivity cells with *z* > 0, and grey scale colour ramp represents unimportant connectivity cells with *z* < 0. Maps were computed using GFlow^49^.

Cell-by-cell correlations in current density values between maps indicated a wide range in concordance between maps varying from a minimum correlation of *r* = 0.34 to 0.94 (Table 2). Redundancy analysis showed that map correlations were most affected by differences in water resistance and scaling function (42.4% and 28.4% of variance explained, respectively; Figs. 4 and 5). In contrast, variation in the *H* index used (*H*_*F*_ vs. *H*_*U*_) only explained 12.5% of the variation and 16.7% of variation among maps could not be explained by marginal effects. Such unexplained variation could be related to specific combinations of factor levels (i.e., interactions) or random variation, e.g., due to random selection of pairs of nodes.

**Table 2:** Cell-by-cell Pearson’s correlation between pairs of maps.

Scaling	Water resistance		High contrast				Low contrast			
			High		Low		High		Low	
		H index	*H*_F_	*H*_U_	*H*_F_	*H*_U_	*H*_F_	*H*_U_	*H*_F_	*H*_U_
**High contrast**	**High**	***H***_**F**_	1.00	**0.79**	*0.73*	0.58	0.83	0.85	0.64	0.67
		***H***_**U**_	**0.79**	1.00	0.41	*0.54*	0.56	0.65	0.34	0.43
	**Low**	***H***_**F**_	*0.73*	0.41	1.00	**0.78**	0.78	0.77	0.91	0.91
		***H***_**U**_	0.58	*0.54*	**0.78**	1.00	0.57	0.64	0.66	0.75
**Low contrast**	**High**	***H***_**F**_	0.83	0.56	0.78	0.57	1.00	**0.94**	*0.85*	0.82
		***H***_**U**_	0.85	0.65	0.77	0.64	**0.94**	1.00	0.78	*0.85*
	**Low**	***H***_**F**_	0.64	0.34	0.91	0.66	*0.85*	0.78	1.00	**0.94**
		***H***_**U**_	0.67	0.43	0.91	0.75	0.82	*0.85*	**0.94**	1.00

The eight maps represent all eight combinations of three factors: scaling function (high or low contrast), water resistance (high or low), and *H* index (*H*_*F*_ and *H*_*U*_). Boxes delineate comparisons using the same scaling function. Bold values denote comparisons between maps that only differ in their *H* index; italic values are comparisons between maps that only differ in their degree of water resistance. (Note: full symmetric matrix is provided for ease of reference).

When using a cut-off (*z* ≥ 0, 1, or 2) to classify cells as important or unimportant for connectivity based on the *z*-score of their current density value, a similar pattern emerged. However, consistencies between map classifications were more affected by scaling function (39.4–47.8%) than water resistance (28.6–33.1%). The effect of variation in the *H* index was considerably smaller, increasing from 1.8% for *z* ≥ 0 to 3.7% for *z* ≥ 2, whereas the proportion of variation unexplained by marginal effects was larger (21.8–27.8%).

Qualitative interpretation of the correlation matrix in Table 2 suggests considerable interactions between factors (which we did not formally include in the db-RDA model to avoid over fitting). For pairs of maps with the same water resistance level, the differences between *H*_*F*_ and *H*_*U*_ maps were more pronounced with the high-contrast scaling (correlation *r* = 0.78 and 0.79) than for the low-contrast scaling (*r* = 0.94). With high-contrast scaling, the effect of water was more pronounced when using the *H*_*U*_ index (*r* = 0.54) than when using the *H*_*F*_ index (*r* = 0.73). With low-contrast scaling, water resistance had a smaller impact on the maps (*r* = 0.85), irrespective of the choice of *H* index.

## Discussion

We presented a conceptual framework for species-agnostic connectivity modelling (Fig. 1) and applied it to model connectivity as current density across the entire province of Alberta, Canada. We assessed the effect of conceptual and computational decisions involved in implementing the approach, and provided a much-needed direct comparison of the degree of uncertainty related to these decisions. First, we examined the effect of the conceptualization of human modification (*H* index) represented by the degree of physical footprint *H*_*F*_ and the intensity of human use *H*_*U*_ on current density maps. Second, we analysed the impact of the scaling function that was used to convert *H* values to resistance, and therefore the contrast between cells with low, intermediate, or high degrees of human modification in their ability to constrain current flow. Thirdly, we assessed the effect of the conceptualization of water bodies as a near-complete barrier on the resulting current density maps. Lastly, we evaluated whether the sensitivity of current density maps to these three parameters is affected by the cut-off threshold used to identify cells that contribute to larger-scale connectivity. We discuss the importance of these factors in an applied land-use planning context.

Since resistance values commonly rely on arbitrary definition by expert opinion, connectivity modelling is often criticized for human decision bias that might affect the resulting maps^23^. In a species-specific approach, expert opinion can be based on a thorough understanding of the life history and ecology of the focal species (e.g.^21^), but key parameters may be unknown or incorrectly assessed. While new optimization methods have been proposed for fitting resistance values based on measures of actual functional connectivity, such as molecular genetic data for a focal species^41^, inference of resistance values has been shown to vary between landscapes even for a single species^63^. The species-agnostic approach relies on the assumption that ecological flow is negatively related to degree of human modification, though this concept is often vaguely defined and may include many dimensions of human modifications. In our study, we conceptualized the degree of human modification for each human-footprint category using two independent parameters, the degree of physical human footprint (*H*_*F*_) and the intensity of its use (*H*_*U*_). An expert panel defined both sets of values, separately for the forest- and agriculture-dominated areas of Alberta. In line with the land-use planning perspective of a species-agnostic modelling of landscape connectivity (Fig. 1b), the assigned values reflect expert consensus about degree of human modification, and not resistance to organismal movement. We believe that such conceptual clarity is important as it makes species-agnostic models more transparent and better justifiable. For the main map, we then used the mean of the two indices *H*_*F*_ and *H*_*U*_. Despite differences in quantitative and relative values of human modifications attributed to human-footprint categories, we found that the resulting current density maps showed limited sensitivity to these variations. Pairwise correlations between maps (based on cell-by-cell current density values) showed a moderate degree of variation between *H*_*F*_ and *H*_*U*_ maps with high-contrast scaling. When a cut-off was used to identify cells that contribute to larger-scale connectivity, however, there was a high degree of consistency between maps. This suggests that the differences may be largely due to variation in the lower end of the distribution of current density values, where we found considerable differences in the shape of distributions (Fig. 3). From an applied perspective, consistency in the upper tail (identification of areas important for larger-scale connectivity) arguably is more important than minor distinctions among areas that contribute relatively little to larger-scale connectivity.

As expected from the literature^38,39^, the scaling method had a major impact on current density maps. We found that a high-contrast scaling amplified the effect of other parameter settings (water resistance, *H* index). High-contrast scaling function increased sensitivity to conceptual decisions regarding the constraining effect of human landscape alterations and topographic features. Such sensitivity is not necessarily bad; rather, large contrasts may be required to effectively model constraints on connectivity. For instance, Koen *et al*.^28^ assigned resistance values of {10, 100, 1000} to natural, unnatural but permeable to movement, and unnatural impermeable land cover types, and successfully validated their current density map for South-eastern Ontario with road mortality data for 20 reptile and amphibian species and with radio-telemetry data for fishers (*Pekania [Martes] pennanti*). As a preliminary step in their study, Dickson *et al*.^34^ compared the impact of different scaling functions on the relative distribution of resistance values (*R*), e.g., by varying the exponent in Eq. 2a. They concluded that small exponents produced exaggerated resistance values for land-use classes with low to intermediate degrees of human modification, and that an exponent of 10 better matched their expectations (Eq. 2a). We used the function used by Dickson *et al*.^34^ in Eqs. 2a and 2b as an upper limit of the range to be considered (high contrast scaling), and we used it for the main map. This scaling gives much more weight to high *H* values than intermediate or low values: for *H* = {0, 0.5, 1}, the function (*H* = 1)^10^ returns the values {1, 57.7, 1024}, with slightly more contrast than Koen *et al*.^38^. We used the linear function in Eq. 2c as a conservative lower limit of scaling functions (low contrast scaling), as it does not affect the relative position of resistance values among human-footprint categories: for *H* = {0, 0.5, 1}, the function (1 + *H* *1000) returns the values {1, 501, 1001}.

Connectivity modelling requires a careful consideration of not only humanmodification type and degree of intensity, but also of natural landscape features that could form potential barriers to movement. We largely followed Dickson *et al*.^34^ in assigning resistance values for water bodies and slope, and Koen *et al*.^28^ likewise treated the resistance of water bodies as barriers equivalent to impermeable non-natural landscape features. However, this is a move away from the conceptually ‘clean’ modelling of landscape connectivity as a function of human modification. Assigning resistance values to natural landscape features implies assumptions about organism movement ability and thus a taxonomic reference. Inevitably, examples can be found where a natural topographic feature may act as a barrier for one species but not for another, thus crossing over from a pure species-agnostic approach to an implicitly multi-species approach (Fig. 1).

Our sensitivity analysis showed a very large effect of the resistance value attributed to water on the resulting current density maps. Based on pairwise correlations between maps, the variation due to resistance to water was considerably larger than the variation due to scaling function. (Note that we did not vary the parameters for resistance due to slope, but its range is more limited, where a slope of 100% results in an increase of resistance values by 25 only). Given the magnitude of the effect of water resistance, the lack of awareness of its importance in previous studies is surprising. For the main map, we thus deviated from Dickson *et al*.^34^ and used an intermediate resistance value for water.

Studies that apply circuit theory to model landscape connectivity are lacking an agreement on a cut-off to distinguish cells that are contributing to the large-scale connectivity. In our study we explored the sensitivity of our maps to the change in three cut-offs represented as *z*-scores for standardized current density values. The shape of the lower tail of the distribution of current density values varied considerably between maps. The proportion of cells with standardized current density values of *z* > 1 (or *z* > 2), however, was relatively constant, but more variable for cells with *z* > 0. Map comparison with db-RDA showed that overall, cell classification was surprisingly robust to the definition of human modification (*H*_*F*_ vs. *H*_*U*_), which becomes slightly more important at higher cut-offs. Taken together, these results indicate that a cut-off of *z* > 1, as suggested by Bowman (*pers. comm*.), may be a robust threshold for identifying cells that are contributing to connectivity.

Species-agnostic approaches for modelling landscape connectivity have been criticized mainly for not being based on species traits. We argue that the power of such species-agnostic connectivity models relies on their generality and direct link to multifunctional land-use planning, as *H* values are assigned to policy-relevant human-footprint categories^28^. As we indicated in our conceptual framework, species-agnostic models are not meant to replace species-specific models designed explicitly to serve specific conservational goals, such as species-at-risk management. It is important to acknowledge that even the use of a species-agnostic model implies assumptions about organismal movement, albeit in a generalized way. The decision of how to treat natural elements like water or slope has been discussed above. An implicit assumption is also that human landscape features affect most species in a similar way. However, examples from the literature indicate, e.g., that some species may actively use roads as corridors, whereas for other species, forest may impede movement^64^. Acceptance of species-agnostic models of landscape connectivity will likely depend on validation with independent ecological data, as successfully performed by Koen *et al*.^28^ for a limited set of species and at a smaller spatial scale. While testing the different models is beyond the scope of the present paper, we plan to use the unique biodiversity monitoring data (with 1656 sites spaced 20 km across Alberta; ABMI) and derived species-level distribution models available through the ABMI to test our connectivity maps. This could be done by assessing which proportion of species turnover across large taxonomic and functional groups can be explained by the current density (i.e., value of being near a “biodiversity highway”) that cannot be explained by current species distribution models based on local site conditions and composition-based landscape metrics (including habitat amount). More research is needed to empirically compare the performance of top-down *vs*. bottom-up connectivity modelling for land-use planning purposes, i.e., to ascertain whether overlaying many species-specific models would result in a substantially different general-use connectivity assessment than species agnostic models.

The main goal of a large-scale connectivity map is to identify and visualize ‘biodiversity highways’, or important routes of ecological flow. Note that a large-scale connectivity map is not designed to evaluate connectivity in a network of patches (e.g., a natural heritage system). To evaluate connectivity between two specific patches, considering all possible movement paths by using current density, nodes would have to be placed in the two patches^42^. In a network model, it is possible to quantify the contribution of each patch or link to overall probability of connectivity^65,66^. However, this should not be done based on current density, as such network analysis aims to distinguish between alternative paths, and hence least cost path distances are better suited for this goal. Linkages between pairs of patches can then be modelled probabilistically based on assumptions about organism’s dispersal ability^50^.

Large-scale, high-resolution modelling of current density remains a computational challenge, despite the new, computationally efficient implementation of Circuitscape in Julia^52^ or the alternative implementation in GFlow^49^. Using GFlow on the Niagara supercomputing facility (SciNet HPC Consortium), we still encountered technical limitations that prevented us from modelling current density at a spatial resolution below 100 m across the province. We calculated resistance values at a high-resolution of 10 m to minimize artefacts related to rasterisation of linear features^67,68^. However, the barrier effect of linear features likely was dampened by aggregating resistance within 100 m cells prior to current density modelling.

Land-cover changes due to human activities and climate change are main drivers of land-use planning, and their study is the basis of land system science (as defined in Verburg *et al*.^69^). This discipline focuses on both the drivers and impacts of land-use change as part of global change. Land systems also offer solutions to global change through adaptation and mitigation and can play a key role in achieving a sustainable future for Earth. Recent studies propose to incorporate landscape ecology issues when designing land systems to understand how and why governance impacts human land change decisions and how those land change patterns influence, and are affected by, the underlying ecological processes (e.g.,^70^). Species-agnostic connectivity modelling based on human modification provides a powerful tool in evaluating the effect of human decisions and actions on multi-level ecological processes and dynamics and simulating the effect of alternative future scenarios.

## Methods

### Study area

**Figure 6:**
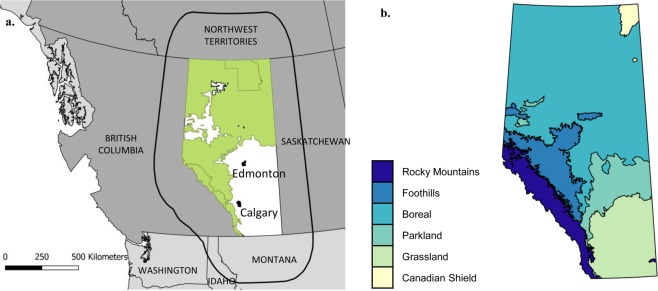
(**a**) Map of the study area encompassing the province of Alberta and neighbouring Canadian Provinces and Territory and US States intersecting with the buffer area (rounded black line surrounding Alberta) around the province. Are represented the forest- (green) and agriculture-dominated (white) areas in Alberta, for which *H* values were allowed to be different for each human-footprint category. **(b)** Delineation of the main natural eco-regions in Alberta.

Our study area, Alberta, is a western Canadian province bordered by British Columbia to the west, Saskatchewan to the east, the Northwest Territories to the north, and Montana (USA) to the south (Fig. 6a). We divided Alberta into two regions (Fig. 6). The forest-dominated area (61% of area), which includes the northern half of the province and the Rocky Mountain area, is mostly covered by native and managed boreal forest and contains most Crown lands and public lands. The agriculture-dominated area (39% of area), which includes the southern half and the central-western part of the province, is dominated by agricultural lands and grasslands and includes most of the urban areas.

### Spatial human footprint data

To map landscape structural connectivity in Alberta as current density, we used ABMI’s Human Footprint Inventory (HFI) derived from the detailed 2014 land-use dataset (for complete information on data collection and computation of the land-use geodatabase, see Geospatial Centre ABMI, 2018). This dataset was derived from 2014 SPOT6 Satellite Imagery (1.5 m Color SPOT 6 Mosaic) provided by Alberta Environment and Sustainable Resource Development, Informatics Branch (https://www.alberta.ca/environment-and-parks.aspx). The original geodatabase consisted of 21 polygon-based layers including 115 land-uses grouped into 6 main categories: agricultural, forestry, transportation, urban, energy, and human-created water bodies^71^.

To avoid boundary effects^72^, we mapped human footprints in a buffer zone surrounding Alberta, using several datasets collected at the federal levels of Canada and the USA. Land cover in the Canadian buffer zone was mapped using raster thematic data originating from classified Landsat 5 and Landsat 7 ortho-images circa 2010 (resolution: 30 meters)^73^. The physical footprint of roads and railways in the Canadian part of the buffer zone was mapped using the National Road and Railways Network databases^74,75^. Land cover in the USA buffer zone was characterized using the National Land Cover Database 2011 for the conterminous United States (NLCD^76^; www.mrlc.gov). The physical footprint of roads and railways in the USA was mapped using TIGER 2014 data (http://www.census.gov/geo/maps-data/data/tiger-line.html). After homogenizing and grouping variables for each dataset, we retained 84 human-footprint categories in total, with 66 categories for Alberta, 8 for the Canadian buffer zone, and 10 for the USA (Appendix 1).

### Mapping landscape resistance

We modified the methodology by Theobald^33^ and Dickson *et al*.^34^ to map landscape resistance and connectivity across Alberta based on the degree of human modification, *H*. At a given location, *H* is the sum of the individual degrees of human modifications *h* caused by all human-footprints at this location^33^. As a major modification of the method, the value of *H* attributed to each human-footprint was conceptualized as the combination of two parameters: degree of physical footprint and intensity of human use (Appendix 1). Values of degree of footprint *H*_*F*_ were based on the degree of change from the native state and the degree of modification. Values of intensity of human use (*H*_*U*_) were based on the assumed average of the amount of human passage, presence, and density throughout the year. *H*_*F*_ and *H*_*U*_ values were assigned summarily to each human-footprint category (not, e.g., for individual roads) and were allowed to differ between the forest- and agriculture-dominated areas of the province (Appendix 1). Both the *H*_*F*_ and *H*_*U*_ indices were calculated on a scale from 0.0 (low footprint, low human use, respectively) to 1.0 (high footprint, high human use, respectively). Values of *H*_*F*_ and *H*_*U*_ indices were assigned based on expert opinion of the Alberta Human Footprint Technical Committee (Alberta Human Footprint Mapping Program). We explicitly asked the experts to focus on the degree of physical footprint and intensity of human use, respectively, without any consideration of particular taxa and their relation with considered human-footprints. The main, average map was calculated as the mean between *H*_*F*_ and *H*_*U*_ indices, hereafter referred to as *H*_*FU*_, whereas for the sensitivity analysis, we used maps with either *H*_*F*_ or *H*_*U*_ but not both.

For each human-footprint *j* (*n* = 84) separately, we attributed an individual value of either 1 or 0 to each 10 × 10 m cell of a raster grid covering the study area, depending on whether or not the cell overlapped with polygons of the considered human-footprint category. We then applied human modification values *h*_*j*_ according to Table 1, separately for *H*_*F*_, *H*_*U*_, or *H*_*FU*_, to cells of value 1. Finally, we combined the 84 layers *h*_*j*_ into a single, overall metric of the degree of human modification *H*, separately for *H*_*F*_, *H*_*U*_, and *H*_*FU*_. In the absence of human footprint, we used a value of *h* = 0. When a cell was impacted by multiple human footprints *j*, we assumed that it should have a higher degree of human modification than a cell with a single human-footprint^33^. We used the fuzzy algebraic sum^77^ for which the result is always at least as large as the largest contributing factor, so that the effect is additive, but never exceeds 1^33^. The degree of human modification *H*_*i*_ at each cell *i* was then calculated, separately for each *H*_*F*_, *H*_*U*_, and *H*_*FU*_, as:

1 $${H}_{i}=1.0-\mathop{\prod }\limits_{j=1}^{k}(1-{h}_{j})$$

where *h*_*j*_ is the degree of modification for an individual human-footprint layer *j* (for *j = 1…k*).

Dickson *et al*.^34^ derived resistance values *R* by rescaling *H* values with a scaling function and adding resistance values for areas with steep slopes *s* and for water bodies *w* (in their case, major rivers), to account for their possible constraining effect on organismal movement:

2a $$R={(H+1)}^{10}+\frac{s}{4}+1000\,\ast \,w$$

In this equation, the maximum resistance due to *H* is (1 + 1)^10^ = 1024, a slope percentage of *s* = 100 carries a penalty of 100/4 = 25, and a water body (*w* = 1) carries a penalty of 1000, which corresponds to a value of *H* = 0.999 (Note that in the rare case of a cell with intensive human modification (*H* = 1) and water body (w = 1), the total resistance could technically reach a value of up to 2024). Percent slope *s* was derived from the US National Elevation Dataset for the entire study area^78^.

We modified Eq. 2a for the main map to assign water bodies a lower resistance value of 57.7, which corresponds to an intermediate value of *H*_*FU*_ = 0.5 (Eq. 2b):

2b $$R={({H}_{FU}+1)}^{10}+\frac{s}{4}+57.7\,\ast \,w$$

For the sensitivity analysis, we independently varied the *H* index (*H*_F_ or *H*_U_), the scaling function, and resistance of water bodies in Eq. 2a as shown in Table 3, resulting in eight combinations. Raising (*H* + 1) to the power of 10 results in a very strong contrast between natural and non-natural landscape features^34^. As a low-contrast alternative, we used a linear scaling function, (1 + H * 1000), which preserves the proportional importance of human modification values. This means that for instance, resistance values *R* based on *H*_F_ with low-contrast scaling of *H* and low resistance of water bodies *w* were calculated as:

2c $$R=(1+{{\rm{H}}}_{F}\,\ast \,1000)+\frac{s}{4}+0\,\ast \,w$$

**Table 3:** Independent factors and levels considered in the sensitivity analysis and their levels, and their associated parameter values.

Factor	Level	Parameter values
H index	*H*_F_	See Table 1
	*H*_U_	See Table 1
Scaling function	Low contrast	(1 + *H* * 1000)
	High contrast	(*H* + 1)^10^
Water resistance	Low	0
	High	1000

Because of computational limitations, we aggregated the 10 × 10 m resolution resistance layers *R* to 100 × 100 m resolution before assessing current density. Cells were aggregated using the mean resistance value of combined 10 × 10 m cells (*aggregate* function in R, *raster* package^79^).

### General procedure for current density computation

We derived a current density map from each resistance map *R* at the 100 × 100 m resolution using GFlow^49^. GFlow is a new software which parallelizes the computation of circuit theory^42^ and allows for simultaneous large-extent and fine-grained connectivity modelling.

We simulated current density between pairs of points (nodes) randomly chosen at each iteration among 50 points that were evenly distributed (approx. every 100 km) along the outer margin of the buffer zone (Fig. 6). This methodology reduces node location bias compared to randomly selecting nodes within the study area and requires fewer pairwise computations^28^. A buffer zone with a linear width of at least 20% of the width of the study area has been shown to be sufficient to remove the effects of node placement on current density^28^. We used the “*Buffer by Percentage”* plugin in QuantumGIS^80^ with a setting of 250% to ensure a sufficient buffer size around Alberta’s polygon (Fig. 6).

To ensure that we considered a sufficient number of random pairs of nodes to converge upon a solution, we set the convergence factor implemented in GFlow (correlation function) to 3 N (=0.999). The convergence value is calculated for each pairwise solve (i.e., iteration) and ranges from 0 to 1. With an increasing number of iterations, the current density maps converge, so that additional connections have a marginally small effect on the result. When the convergence threshold is reached, the computation stops and the current density map is completed^49^. As a final step, we removed the buffer zone to only keep Alberta’s extent.

### Map comparison and evaluation of the uncertainty

We standardized current density values as *z*-scores (*z =* $\bar{x}$*/sd*) by subtracting the mean value $\bar{x}$, and dividing by the standard deviation *sd*. We used three different thresholds (*z* ≥ 0, 1, or 2) for identifying cells with important contribution to connectivity. We assessed the relative contribution of the three factors listed in Appendix 1 to the variance between current density maps in a factorial design with variation partitioning using distance-based redundancy analysis (db-RDA^81^). First, we derived an 8 × 8 correlation matrix by calculating the Pearson correlation *r* between each pair of maps. For each of the three cut-off levels, we derived an 8 × 8 similarity matrix by counting, for each pair of maps, the proportion of cells that were classified the same way (either as ‘important’ or as ‘unimportant’ in both maps). All four matrices were converted to dissimilarity matrices. For each dissimilarity matrix, we performed a db-RDA^82^ where the three explanatory factors (*H* index, scaling function, and water resistance) were coded as dummy variables. We used the function *varpart* in the R package *vegan* to obtain adjusted *R*^2^ values for the contribution of each factor.

Unless otherwise stated, all computations and analyses were performed in R 3.4.2^83^ on Niagara supercomputer at the SciNet HPC Consortium.

## Supplementary information

Appendix 1.

## Data Availability

The full human-footprint dataset of Alberta can be found at https://abmi.ca/home/data-analytics. Dataset and R script used for the ANOVA / statistical analysis is available at 10.5683/SP2/KJVW8Y.
