# Supplementary material for: Conceptual framework and uncertainty analysis for large-scale, species-agnostic modelling of landscape connectivity across Alberta, Canada
Source: Sci Rep. 2020 Apr 22;10:6798. doi: 10.1038/s41598-020-63545-z (PMC7176682; doi:10.1038/s41598-020-63545-z)
Supplement: Supplementary file 1 — Appendix 1. [file 41598_2020_63545_MOESM1_ESM.pdf]

APPENDIX 1

Conceptual framework and uncertainty analysis for large-scale, species-agnostic modelling of landscape connectivity across Alberta, Canada

Authors: Ronan MARREC[1,2]*, Hossam E. ABDEL MONIEM[2,3,4], Majid IRAVANI[5], Branko HRICKO[5], Jahan KARIYEVA[5], Helene H. WAGNER[2]

Affiliations:

[1] EDYSAN (*Ecologie et Dynamique des Systèmes Anthropisés*) UMR 7058 CNRS-Université de Picardie Jules Verne, 33 rue Saint Leu, F-80039 Amiens, France

[2] Department of Ecology and Evolutionary Biology, University of Toronto, Canada

[3] Centre for Urban Environments, University of Toronto Mississauga, Canada

[4] Department of Zoology, Faculty of Science, Suez Canal University, Ismailia, Egypt

[5] Alberta Biodiversity Monitoring Institute, University of Alberta, Canada

*Correspondence and requests for materials should be addressed to R.M. (e-mail: ronan.marrec@u-picardie.fr)

ORCID: R. Marrec, 0000-0003-1607-4939

Appendix 1 List of human footprint (HF) categories considered in the study, listed following the levels of classification proposed by the Conservation Measures Partnership framework[84]. For each HF is given its degree of footprint ($\boldsymbol{H_F}$) and intensity of human use ($\boldsymbol{H_U}$) values. Distinction was made between the forest- (FD) and agriculture-dominated (AD) areas for Alberta's HF only.

**Human modification activities framework**			**FD area**		**AD area**	
**Level 1**	**Level 2**	**HF category**	$\boldsymbol{H_F}$	$\boldsymbol{H_U}$	$\boldsymbol{H_F}$	$\boldsymbol{H_U}$
**Alberta**						
**Residential and commercial development**	Housing and urban areas	Clearing (residence)	0.75	1	0.75	1
		Rural residence	0.5	0.5	0.5	0.5
		Country residence	0.5	0.65	0.5	0.65
		Urban residence	1	1	1	1
	Commercial and industrial areas	Clearing (unknown)	0.6	0	0.5	0
		Clearing (well pad)	0.6	0.2	0.5	0.2
		Industrial facility	1	1	1	1
	Tourism and recreation areas	Green space	0.25	0.5	0.25	0.5
		Runway	0.5	0.3	0.5	0.3
		Surrounding vegetation	0.5	0.5	0.5	0.5
		Recreation	0.25	0.25	0.35	0.25
		Campground	0.5	0.5	0.5	0.5
		Golf course	0.6	0.5	0.65	0.5
**Agriculture and aquaculture**	Annual and perennial non-timber crops	Crop	0.75	0.25	0.85	0.25
	Livestock farming and ranching	CFO	1	1	1	1
		Rough pasture	0.25	0.25	0.35	0.25

		Tame pasture	0.6	0.25	0.7	0.25
**Energy production and mining**	Oil and gas drilling	Oil sand mine	1	1	1	1
		Gas well	0.9	0.25	0.8	0.25
		Oil well	0.4	0.25	0.8	0.25
	Mining and quarrying	Gravel/sand/coal mine (> 15 years)	0.5	0.5	0.5	0.5
		Gravel/sand/coal mine (15-10 years)	0.8	0.8	0.8	0.8
		Gravel/sand/coal mine (< 10 years)	1	1	1	1
		Pit lake	1	0.1	1	0.1
		Peat	0.8	1	0.8	1
		Mine (RIS)	1	1	1	1
		Well (RIS)	1	1	1	1
		Well active	0.4	0.25	0.8	0.25
		Well abandoned	0.1	0	0.2	0
	Renewable energy	Windmill	1	0.1	1	0.1
**Transportation and service corridors**	Roads and railroads	Gravel road	0.8	0.75	0.8	0.75
		Unpaved road	0.7	0.3	0.7	0.3
		Winter access road	0.4	0.2	0.4	0.2
		Trail	0.1	0.2	0.2	0.3
		Paved road	1	1	1	1
		Unimproved road	0.4	0.25	0.5	0.3
		Abandoned railway	0.6	0	0.6	0
		Single track railway	0.9	0.25	0.9	0.25
		Double track railway	0.9	0.4	0.9	0.4
		Multiple railway and spur	0.9	0.6	0.9	0.6
	Utility and service lines	Transmission line (RIS)	1	1	1	1

		Transmission line	0.3	0.25	0.6	0.1
		Pipeline	0.65	0.65	0.65	0.65
		Pipeline (RIS)	1	1	1	1
		Low-impact seismic line	0	0.1	0.1	0
		Pre-low-impact seismic line	0	0.1	0.6	0.1
		Seismic line trail	0.1	0.1	0.25	0.2
	Logging and wood harvesting	Cut block (> 15 years)	0	0	0	0
		Cut block (15-4 years)	0.6	0.5	0.6	0.5
		Cut block (< 4 years)	1	0.7	1	0.7
	Dams and water management/use	Reservoir	1	0.25	1	0.25
		Canal	1	0.1	1	0.15
	Other ecosystem modifications	Borrow pit dry	0.8	0.1	0.8	0.1
		Borrow pit wet	1	0.1	1	0.1
		Dugout	1	0.25	1	0.25
		Borrow pit (RIS)	1	1	1	1
		Railway/road verge	0.5	0.1	0.5	0.1
		Disturbed vegetation	0.7	0.5	0.7	0.5
		Windrow (RIS)	1	1	1	1
**Pollution**	Household sewage and urban waste water	Lagoon	1	0.15	1	0.15
	Industrial and military effluents	Sump	1	0.2	1	0.2
		Tailing pond	1	0.5	1	0.5
		Tailing pond (RIS)	1	1	1	1
	Garbage and solid waste	Landfill	1	1	1	1
		Transfer station	1	0.45	1	0.45
		Mine wastes	1	1	1	1
**Canada outside of Alberta**						
**Transportation and service**	Roads and railroads	Gravel road	0.8	0.75	-	-
		Unpaved road	0.7	0.3	-	-

**corridors**		Winter access road	0.4	0.2	-	-
		Paved road	1	1	-	-
		Railway	0.9	0.5	-	-
**Agriculture and aquaculture**	Annual and perennial non-timber crops	Crop	0.75	0.25	-	-
	Livestock farming and ranching	Tame pasture	0.6	0.25	-	-
**Other**	Mixed use development	Settlement	0.75	0.8	-	-
**USA**						
**Transportation and service corridors**	Roads and railroads	Gravel road	0.8	0.75	-	-
		Paved road	1	1	-	-
		Railway	0.9	0.5	-	-
**Agriculture and aquaculture**	Annual and perennial non-timber crops	Crop	0.5	0.4	-	-
	Livestock farming and ranching	Rough pasture	1	1	-	-
		Tame pasture	0.5	0.65	-	-
**Other**	Mixed use development	Disturbed vegetation	0.5	0.5	-	-
		Development high intensity	0.75	0.25	-	-
		Development medium intensity	0.25	0.25	-	-
		Development low intensity	0.6	0.25	-	-

RIS: Reclamation Information System data obtained from the Government of Alberta, Alberta Environment and Parks. These land uses are found in the oil-sand region surrounding Fort McMurray and considered as more intensive than similar land uses elsewhere in Alberta.
